# Inhibition of GABAergic Neurons and Excitation of Glutamatergic Neurons in the Ventrolateral Periaqueductal Gray Participate in Electroacupuncture Analgesia Mediated by Cannabinoid Receptor

**DOI:** 10.3389/fnins.2019.00484

**Published:** 2019-05-17

**Authors:** He Zhu, Hong-Chun Xiang, Hong-Ping Li, Li-Xue Lin, Xue-Fei Hu, Hong Zhang, Wang-Yang Meng, Lu Liu, Chao Chen, Yang Shu, Ru-Yue Zhang, Pei Zhang, Jun-Qiang Si, Man Li

**Affiliations:** ^1^Department of Neurobiology and Key Laboratory of Neurological Diseases of Ministry of Education, School of Basic Medicine, The Institute of Brain Research, Tongji Medical College of Huazhong University of Science and Technology, Wuhan, China; ^2^Department of Physiology, Medical College of Shihezi University, Shihezi, China; ^3^Department of Central Laboratory, Affiliated Hospital of Jiangsu University, Zhenjiang, China

**Keywords:** pain, electroacupuncture, chemogenetics, GABAergic neuron, glutamatergic neuron, vlPAG

## Abstract

Although electroacupuncture (EA) has become a worldwide practice, little is understood about its precise target in the central nervous system (CNS) and the cell type-specific analgesia mechanism. In the present study, we found that EA has significant antinociceptive effects both in inflammatory and neuropathic pain models. Chemogenetic inhibition of GABAergic neurons in the ventrolateral periaqueductal gray (vlPAG) replicated the effects of EA, whereas the combination of chemogenetic activation of GABAergic neurons and chemogenetic inhibition of glutamatergic neurons in the vlPAG was needed to reverse the effects of EA. Specifically knocking out CB1 receptors on GABAergic neurons in the vlPAG abolished the EA effect on pain hypersensitivity, while specifically knocking out CB1 receptors on glutamatergic neurons attenuated only a small portion of the EA effect. EA synchronously inhibits GABAergic neurons and activates glutamatergic neurons in the vlPAG through CB1 receptors to produce EA-induced analgesia. The CB1 receptors on GABAergic neurons localized in the vlPAG was the basis of the EA effect on pain hypersensitivity. This study provides new experimental evidence that EA can bidirectionally regulate GABAergic neurons and glutamatergic neurons via the CB1 receptors of the vlPAG to produce analgesia effects.

## Introduction

A series of randomized controlled trials of acupuncture and analgesia have shown that acupuncture has an analgesic effect in the context of chronic pain, such as knee pain, low back pain, migraine, and fibromyalgia (Liu et al., 2018; Mist and Jones, 2018; Musil et al., 2018). However, the specific target sites in the central nervous system (CNS) for electroacupuncture (EA) analgesia are still unclear, which seriously hinders the clinical promotion and application of EA analgesia.

Studies have confirmed that EA can induce the specific expression of c-fos in the periaqueductal gray (PAG) region (Fusumada et al., 2007). It has been demonstrated that the ventrolateral periaqueductal gray (vlPAG) is an essential part of the neural pathway that mediates pain regulation (Ho et al., 2011). Microinjection of GABA agonists into the vlPAG promotes pain, and microinjection of GABA antagonists produces antinociceptive effects by reducing inhibitory neurotransmission (Bobeck et al., 2014; Takasu et al., 2015). According to the GABA disinhibition hypothesis, tonically active GABAergic interneurons are present within the PAG, which release the neurotransmitter GABA that acts via GABA_A_ receptors to inhibit spinally projecting output neurons (Basbaum and Fields, 1984). It was proposed that opioids and cannabinoids activate the descending pathway by indirectly suppressing the inhibitory influence of local GABAergic interneurons, thereby disinhibiting the antinociceptive pathway of the neuronal output to the spinal cord (Meng et al., 1998).

The endocannabinoid system, which is a crucial neuromodulatory system involved in the control of pain transmission and acupuncture analgesia within the CNS (Chen et al., 2009). Cannabinoid-induced antinociception plays a role in the activation of a descending inhibitory pain pathway (Palazzo et al., 2010). Cannabinoid receptor 1(CB1) are expressed in both nerve endings of GABAergic neurons and glutamatergic neurons in PAG and activation of the CB1 receptor may regulate GABAergic and glutamatergic neurotransmission (Drew et al., 2008; Tjen-A-Looi et al., 2009). Our previous results have shown that EA reversed the reduced expression of CB1 receptors and the 2-arachidonoylglycerol (2-AG) level in the midbrain in chronic pain conditions (Yuan et al., 2018). These results indicated that EA may exert an analgesic effect by acting on the CB1 receptor. Microinjection of the CB1 receptor antagonist AM251 into the vlPAG can reverse the EA effect on pain hypersensitivity, which further confirms the mechanism of EA exerting an antinociceptive effect through CB1 receptors in the vlPAG (Yuan et al., 2018). Despite the abundant evidence supporting the hypothesis that the CB1 receptor is involved in EA analgesia, the exact cell type involved in EA analgesia through vlPAG descending pain modulation has not been directly investigated. In this study, with the help of cell type-specific chemogenetic manipulations in the vlPAG, we attempt to verify the hypothesis that GABAergic neurons and glutamatergic neurons are involved in CB1-mediated EA antinociception.

## Materials and Methods

### Animals

All animal experiments were ratified by the Animal Care and Use Committee of Huazhong University of Science and Technology, the procedures conform to the ethical guidelines of the International Association for the Study of Pain (Demers et al., 2006). Eight-weeks-old male C57BL/6 mice (20–25 g) were obtained from Beijing Vital River Laboratory Animal Technology Co., Ltd. The mCnr1flox/flox mice were bought from the Cyagen biosciences laboratory (Nanjing). GAD67-GFP mice were kindly provided by Dr. Xiangning Li (Huazhong University of Science and Technology, Whuhan, China). The mice were individually housed in cages with a 12-h light/dark cycle and had free access to food and water. The mice used for EA experiments in CCI and KOA mice were divided into four groups (control, CCI or KOA, EA, and sham EA). The mice used for chemogenetic manipulation and knockout experiments in CCI and KOA mice were divided into three groups (control, chemogenetic manipulation or knockout, EA).

### Viruses Constructs and Surgery

Adeno-associated viruses (AAV/2-9) were designed to achieve CRE-DIO system-mediated chemogenetic manipulation strategy: rAAV-mDlx-CRE-WPRE-pA combined with rAAV-hSyn-DIO-hM3D(Gq)-mCherry-WPRE-pA or rAAV-hSyn-DIO-hM4D(Gi)-mCherry-WPRE-pA were designed to excite or inhibit GABAergic neurons of vlPAG (Dimidschstein et al., 2016). rAAV-hSyn-mCherry-WPRE-pA was selected as control virus. Furthermore, rAAV-CaMKIIa-HA-KORD-IRES-mCitrine-WPRE-pA, selecting salvinorin B as DREAD, could inhibit glutaminergic neurons of vlPAG individually on the basis of above combined operations and without interactions.

All viruses used in this study were acquired from the Wuhan BrainVTA scientific and technical corporation. Before surgery, mice were anesthetized with isoflurane and fixed in the stereotaxic apparatus (RWD Instruments,China). Make a 1.5 cm length longitudinal incision along the midline of the skull, gently remove the periosteum from the exposed surface of the surgical area. Viruses injections were performed using the coordinates of vlPAG as following: −4.8 mm from bregma, −0.4 mm lateral from midline, and 2.8 mm ventral to skull. Desired viruses vectors (150 nL) were injected into the vlPAG at a rate of 50 nl per 60 s (Samineni et al., 2017). If the virus infection area exceeds the vlPAG area, it is not included in the statistics (Figure S5).

### Chemogenetic Manipulation

Three weeks after viruses injections, mice were intraperitoneally injected with clozapine N-oxide (CNO, Sigma) 60 min before the behavioral assessment. All baselines for thermal and mechanical sensitivity were recorded after the viruses injections and before the CNO administration. We administered 1 mg/kg CNO for both hM3Dq activation and hM4Di inhibition (Samineni et al., 2017). In rAAV-CaMKIIa-HA-KORD-IRES-mCitrine-WPRE-pA operation mice, we individually administered 5 mg/kg salvinorin B for inhibiting glutaminergic neurons of vlPAG even on the basis of GABAergic neurons have been excited by hM3Dq activation with CNO (Vardy et al., 2015). In the chemogenetic reversing experiment of EA antinociception, CNO was applied just before EA treatment.

### CCI Model

Neuropathy was induced by chronic constriction injury (CCI) of the sciatic nerve using a similar procedure for rats (Bennett and Xie, 1988) which was adapted for mice (Sommer et al., 1998). Two loosely constrictive ligatures were tied around the left sciatic nerve to slow the blood flow of the sciatic myelin blood vessels without causing acute crushing damage. The sham group animals were anesthetized, their sciatic nerves were only exposed without constriction. Sham-operated animals were used as neuropathy controls.

### Induction of Knee Osteoarthritis (KOA)

Intra-articular injection of monosodium iodoacetate (MIA) (Sigma, UK) into the left knee joint was applied to induce KOA model. Five microliters of 5 mg/ml MIA in sterile saline (0.9%) were injected into the joint space of the left knee through the infrapatellar ligament with a 30-gauge needle (La Porta et al., 2013). This method causes histopathology changes in the cartilage (van Osch et al., 1994) and produces obvious joint pain in mice (Harvey and Dickenson, 2009). The sham-operated mice received an intra-articular injection of 5 μl of 0.9% sterile saline.

### EA Treatment

For the CCI model EA treatment group, the mouse received EA on the left “Huantiao” (GB30) and “Yanglingquan” (GB34) once a day, starting from the eighth day after operation. GB30 and GB34 were chosen based on their effect in improving inflammatory pain and neuropathic pain in mouse (Kang et al., 2007; Park et al., 2014; Lee et al., 2018). Two acupuncture needles were inserted 2–3 mm deep into two acupoints corresponding to GB30 and GB34 in humans. EA (1 mA and 0.1 ms) was carried out at 2 Hz for 30 min (Wu et al., 2013). Current was delivered with a modified constant current Han's Acupoint Nerve Stimulator (LH202, Huawei Co. Ltd., Beijing, China) (Wu et al., 2013).

For the EA treatment group of KOA, mice received EA administration on the left “Neixiyan” (Ex-LE4) and “Dubi” (ST35) for 7 days, starting from the fifteenth day after MIA injection. EA (1 mA and 0.1 ms) was carried out at 2 Hz for 30 min. Ex-LE4 and ST35 were chosen based on the fact that their using frequency is the highest in KOA (Wu et al., 2010).

For sham treatment control, acupuncture needles were inserted into above acupuncture points without electrical stimulation or manual manipulation. Previous studies showed that needles inserted into active acupoints, but no electrical or manual stimulation, do not produce analgesia (Lao et al., 2004). During EA treatment, each mouse was placed in a homemade bag. Control group also received the same method to exclude the stress response. The animals remained still during EA treatment and showed no evident signs of distress.

### Nociceptive Behavioral Tests

Mechanical allodynia and heat hyperalgesia were checked with von Frey filaments and the hot plate, respectively (Fernihough et al., 2004). The animals were habituated to the testing environment for 30 min. The behavioral tests were performed 3 times before model induction and every other day after model induction until the formal experimental operation. The behavioral tests were performed once a day during kinds of experimental operations (Figure S1).

The surface temperature of the hot-plate was maintained at 53°C. The withdrawal latency started from the mouse was put on the plate and terminated when a quick/prolonged withdrawal or flick of the paw was observed. Twenty seconds was set as a cut-off time for mice to prevent tissue damage (Chen et al., 2017). Thermal stimuli were delivered three times to hindpaw at 10 min intervals, and the mean value was calculated.

The tactile withdrawal threshold of mice was measured by using the “up-down” method (Chaplan et al., 1994). After an acclimation period of 30 min, we stimulated the plantar surface of the hindpaw vertically with a series of von Frey (Stoelting, Wood Dale, IL) hairs with logarithmically increasing stiffness, and bent the filament for 5 s to the central plantar surface with sufficient force. Brisk withdrawal or paw flinching was considered as a positive response. The test of tactile withdrawal threshold was repeated two times in each mouse, and the mean value was calculated.

### Conditioned Place Preference (CPP) Test

The test trials consisted of one habituation (1st day), four conditionings (2nd−5th days), and one test (6th day) (Hnasko et al., 2005). In the case of EA treatment, the mice were confined in white box, thereby achieving EA treatment and white box condition matching. On the test day, animals were free to go any parts of the apparatus, and the time spent in bright or dark box was measured. Data were analyzed by the SuperMaze software (Xinsoft SuperMaze Animal Behavior Analysis System, Shanghai).

### Immunofluorescence

Mice under deep anesthesia with 10% chloralic hydras were transcardially perfused with 37°C normal saline followed by 4% paraformaldehyde in 0.1 M PBS (pH, 7.4; 4°C). The brain were removed immediately and post-fixed in the same fixative. Then, the tissues were cryoprotected in 20 and 30% sucrose in 0.1 M PBS for 24 h, respectively, at 4°C. The OCT embedded blocks were sectioned for 25-μm thickness. Sections from each group were rinsed in 0.01 M PBS and blocked for 2 h with blocking liquid (5% donkey serum and 0.2% tween-20 in 0.01 M PBS) at room temperature. The sections were probed with the following antibodies: rabbit anti-CB1 (1:200), mouse anti–GAD67 (1:200), guinea pig anti-VGLUT2 (1:100). Subsequently, the free-floating sections were washed with 0.01M PBS 3 times, and incubated with following secondary antibodies (Jackson ImmunoResearch) for 2 h: donkey anti-rabbit IgG conjugated with Dylight 594 (1:600), donkey anti-mouse IgG conjugated with Dylight 488 (1:600), donkey anti-guinea pig IgG conjugated with Dylight 488 (1:400). The sections were incubated with DAPI for the nucleus staining for 8 min, and washed 3 times in 0.01M PBS and then cover-slipped. The samples were studied under a fluorescence microscope (Olympus) for the immunefluorescence staining. Images were analyzed by using NIH Image J software (Bethesda, MD, USA). The layouts of images were based on the Photoshop (ADOBE company, USA).

### Statistics

Results of data are expressed as mean ± SEM. The thermal latency and withdrawal thresholds between different groups over time were tested with two-way analysis of variance (ANOVA) followed by Bonferroni *post hoc* tests. Other data were analyzed by One-way ANOVA and Newman-Keuls *post hoc* test (SPSS, Version 11.0). Results represented as significance based on a value of *P* < 0.05.

## Results

### EA Effectively Reduces Pain Hypersensitivity in CCI and KOA Mice

CCI or KOA induction significantly reduced mechanical withdrawal thresholds and thermal withdrawal latencies (Figures S2A–D).

**Figure 1:**
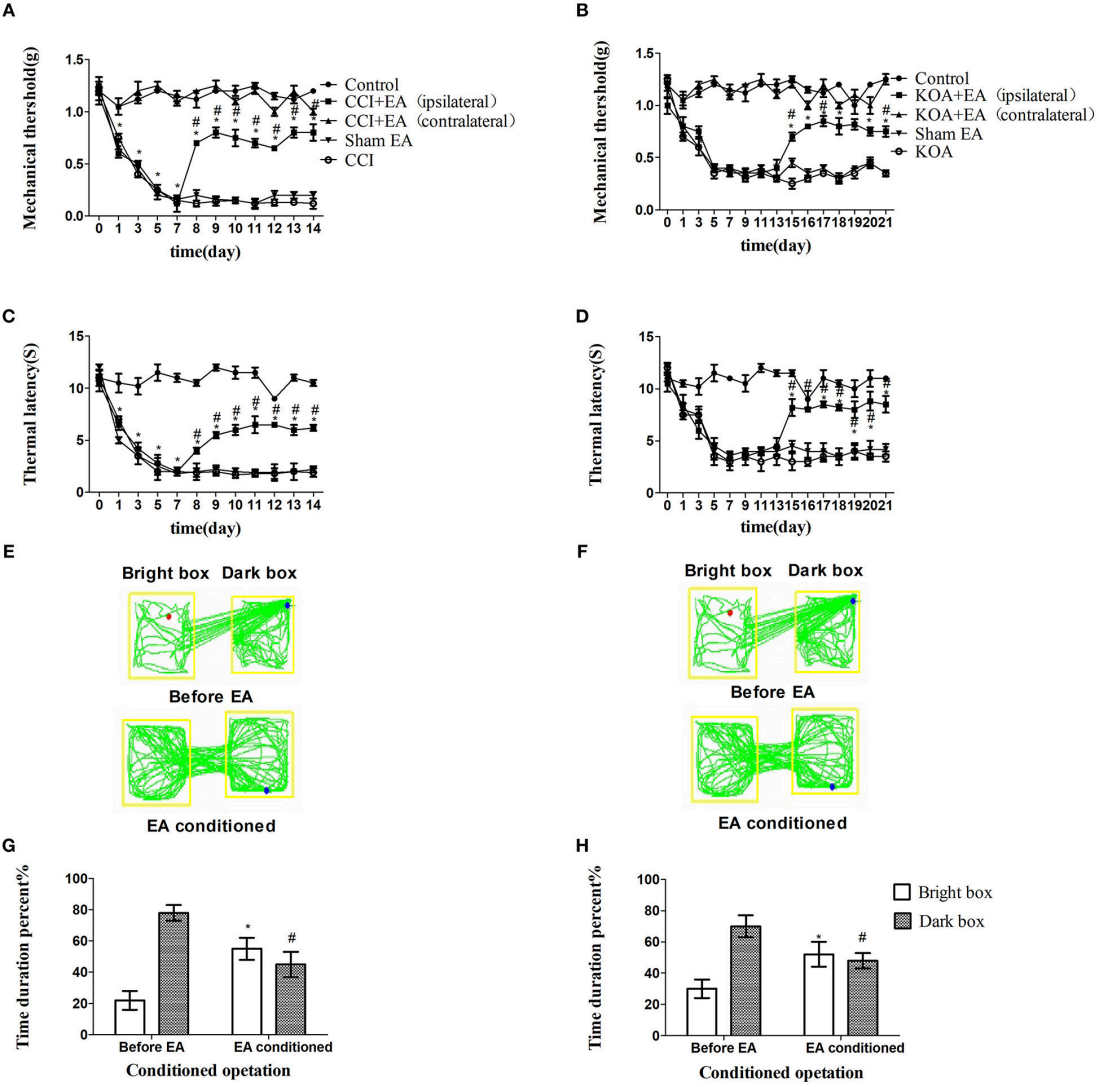
EA effectively reduces pain hypersensitivity in CCI and KOA mice. **(A,B)** Time course of tactile threshold in response to von Frey filaments. **(C,D)** The noxious heat stimulus (53°C) caused a change in the thermal thresholds. The effect of tactile **(A)** and thermal **(C)** withdrawal thresholds changes in CCI mice. The change of tactile **(B)** and thermal **(D)** withdrawal thresholds of KOA mice caused by EA. A motion map of the EA-conditioned CPP test in CCI **(E)** and KOA mice **(F)**. **G,H** are summary values of motion trajectories. EA (1 mA and 0.1 ms) at 2 Hz was administered for 30 min, once a day starting from the 8th to the 14th day in the CCI model and starting from the 15th to 21st day in the KOA model. Control group consisted of sham-operated mice. Data are expressed as the means ± SEM (*n* = 12 in each group). In **A–D**, ^*^p < 0.05, compared with the control group; ^#^*p* < 0.05, compared with the model group; In panels **G,H**,^*^p < 0.05, compared with the bright box time duration percent before EA; ^#^*p* < 0.05, compared with the dark box time duration percent before EA.

The tactile threshold and thermal withdrawal latency were effectively increased by EA in CCI and KOA mice (Figures 1A–D).

The repeated EA treatment also affected the behavior in the CPP test, as the mouse spent more time in the bright box after being paired with EA treatment (Figures 1E–H).

### Effect of Chemogenetic Inhibition of GABAergic Neurons in vlPAG

Since the PAG is a vital brain region related to the descending pain control system, both cannabinoids and opioids, released by EA, may inhibited GABAergic neurons to produce antinociceptive effect by GABAergic disinhibition mechanism (Osborne et al., 1996; Finn et al., 2003), we applied a chemogenetic strategy to assess this hypothesis.

**Figure 2:**
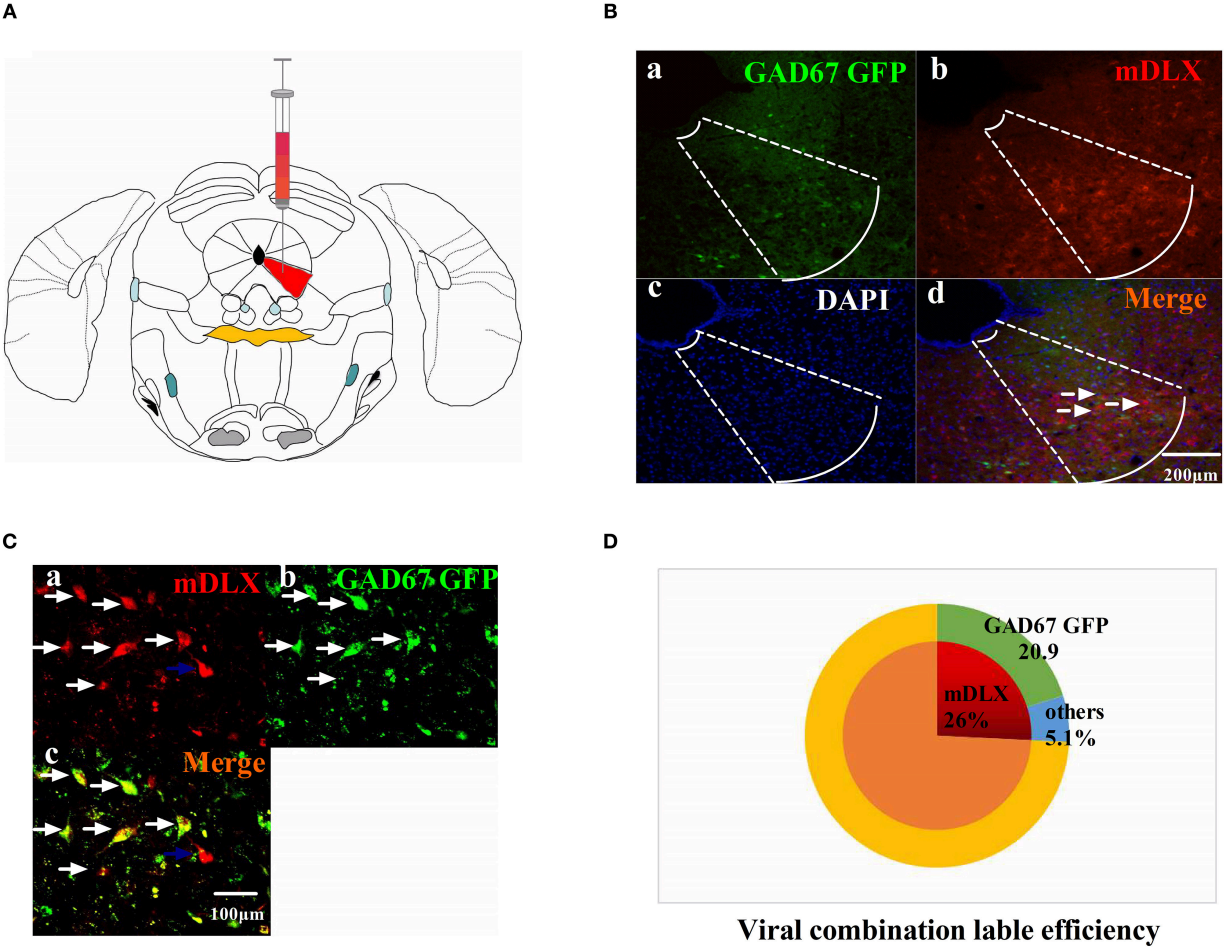
The viruses combination cocktail label efficiency. **(A)** Pattern map of the viruses injection area. The red color region is the vlPAG. **(B)** The viruses combination cocktail was injected into the right side of the vlPAG of GAD67 GFP mice. (a) GAD67 GFP cells in the vlPAG (green). (b) Viruses-labeled cells (red). (c) DAPI nuclear staining (blue). (d) Colocalization fluorescence (pink), arrows show typical fluorescent, colocalized cells. Scale bar, 200 μm. **(C)** High magnification images, scale bar, 100 μm. White arrows indicate cells that are co-labeled, and blue arrows indicate cells that are mDLX-labeled but not labeled with GAD67 GFP. **(D)** Summary data show the percentage of colocalized cells in the area of total viruses-labeled cells. Red sector, viruses-labeled cells; green sector, GAD67 GFP cells; blue sector, viruses-labeled other cells. Data are expressed as the means ± SEM (*n* = 3 mice in each group).

The viruses combination cocktail of rAAV-mDlx-CRE-WPRE-pA and rAAV-hSyn-DIO-hM3D(Gi)-mCherry-WPRE-pA, designed to inhibit GABAergic neurons, was injected into the right side of the vlPAG (Figure 2A). The viruses combination was injected into the right side of the vlPAG of GAD67 GFP mouse to confirm the viruses combination efficiency (Figures 2B,C). A total of 80.5% of the viruses combination-tagged neurons in the vlPAG were marked together with GAD67 GFP-positive cells (Figure 2D).

**Figure 3:**
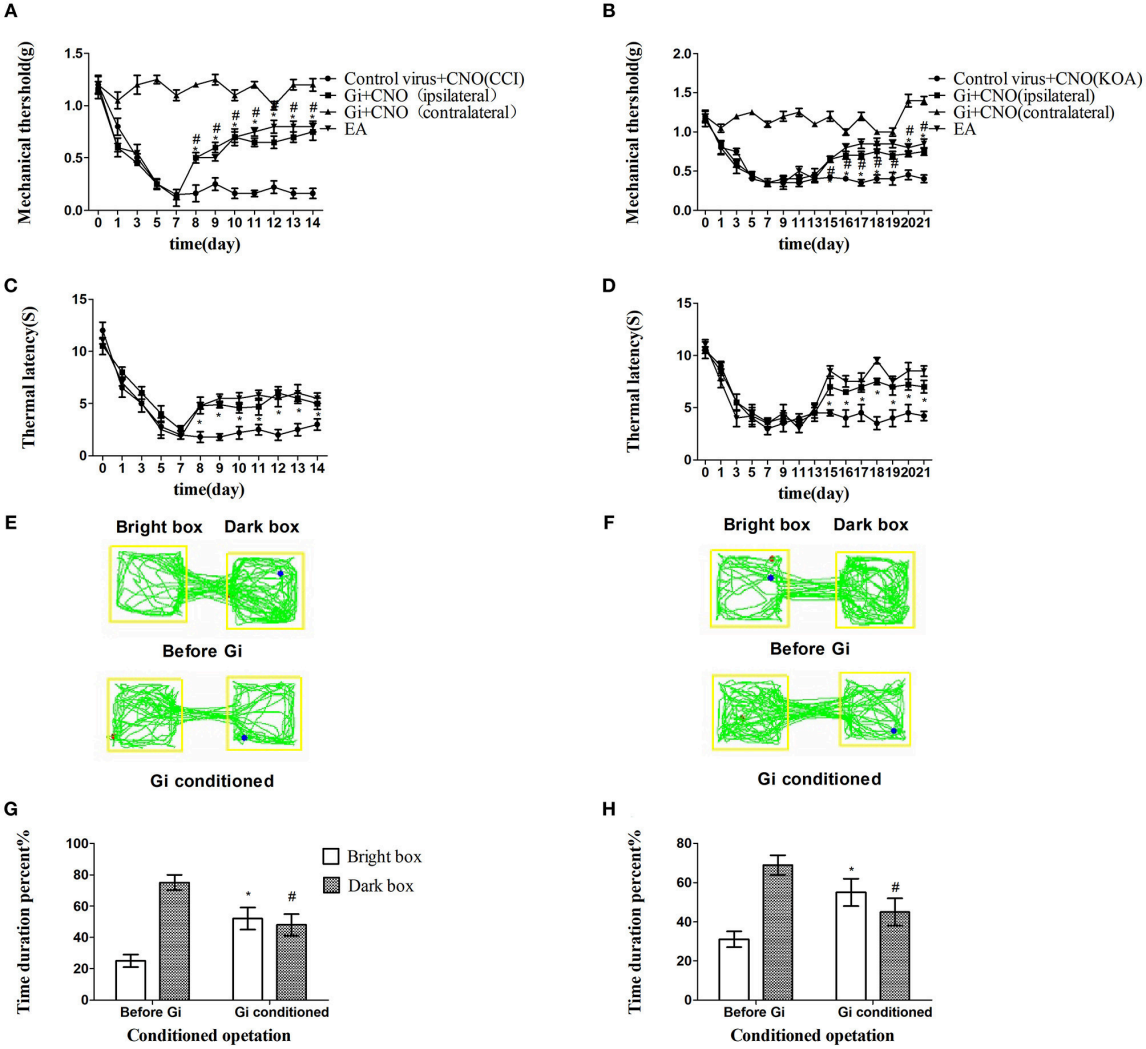
Chemogenetic inhibition of GABAergic neurons in the vlPAG replicated the effects of EA. **(A,B)** Time course of tactile threshold in response to von Frey filaments. **(C,D)** The noxious heat stimulus (53°C) caused a change in the thermal thresholds. Tactile **(A)** and thermal **(C)** withdrawal thresholds changes in CCI mice after chemogenetic inhibition. Changes in tactile **(B)** and thermal **(D)** withdrawal thresholds of KOA mice caused by chemogenetic inhibition. Motion map of chemogenetic inhibition of conditioning in the CPP test with CCI **(E)** and KOA mice **(F)**. **G,H** are summary values of motion trajectories. Virus was injected 21 days before the behavior test. CNO (1 mg/kg) was administered once a day starting from the 8th to the 14th day in the CCI model and starting from the 15th to the 21st day in the KOA model. EA (1 mA and 0.1 ms) at 2 Hz was administered for 30 min. Once a day starting from the 8th to the 14th day in the CCI model and starting from the 15th to the 21st day in the KOA model, all behavior tests were completed 2 h after CNO administration. Control groups consisted of CCI or KOA mice injected with control virus and CNO but without EA treatment. Data are expressed as means ± SEM (*n* = 12 in each group). In **A–D**, ^*^p < 0.05, compared with control group; ^#^*p* < 0.05, compared with the model group; in **G,H**,^*^p < 0.05, compared with the bright box time duration percent before chemogenetic inhibition; ^#^*p* < 0.05, compared with the dark box time duration percent before chemogenetic inhibition.

Chemogenetic inhibition of GABAergic neurons reliably replicated the effect of EA, including the antinociceptive effects (Figures 3A–H).

### Chemogenetic Activation of GABAergic Neurons in vlPAG Only Partly Attenuated the Effect of EA

On the basis of chemogenetic inhibition of GABAergic neurons reliably replicating the antinociceptive effect of EA, we considered if chemogenetic activation could abolish the effect of EA. The viruses combination of rAAV-mDlx-CRE-WPRE-pA and rAAV-hSyn-DIO-hM3D(Gq)-mCherry-WPRE-pA, designed to activate GABAergic neurons, was injected into the right side of the vlPAG.

**Figure 4:**
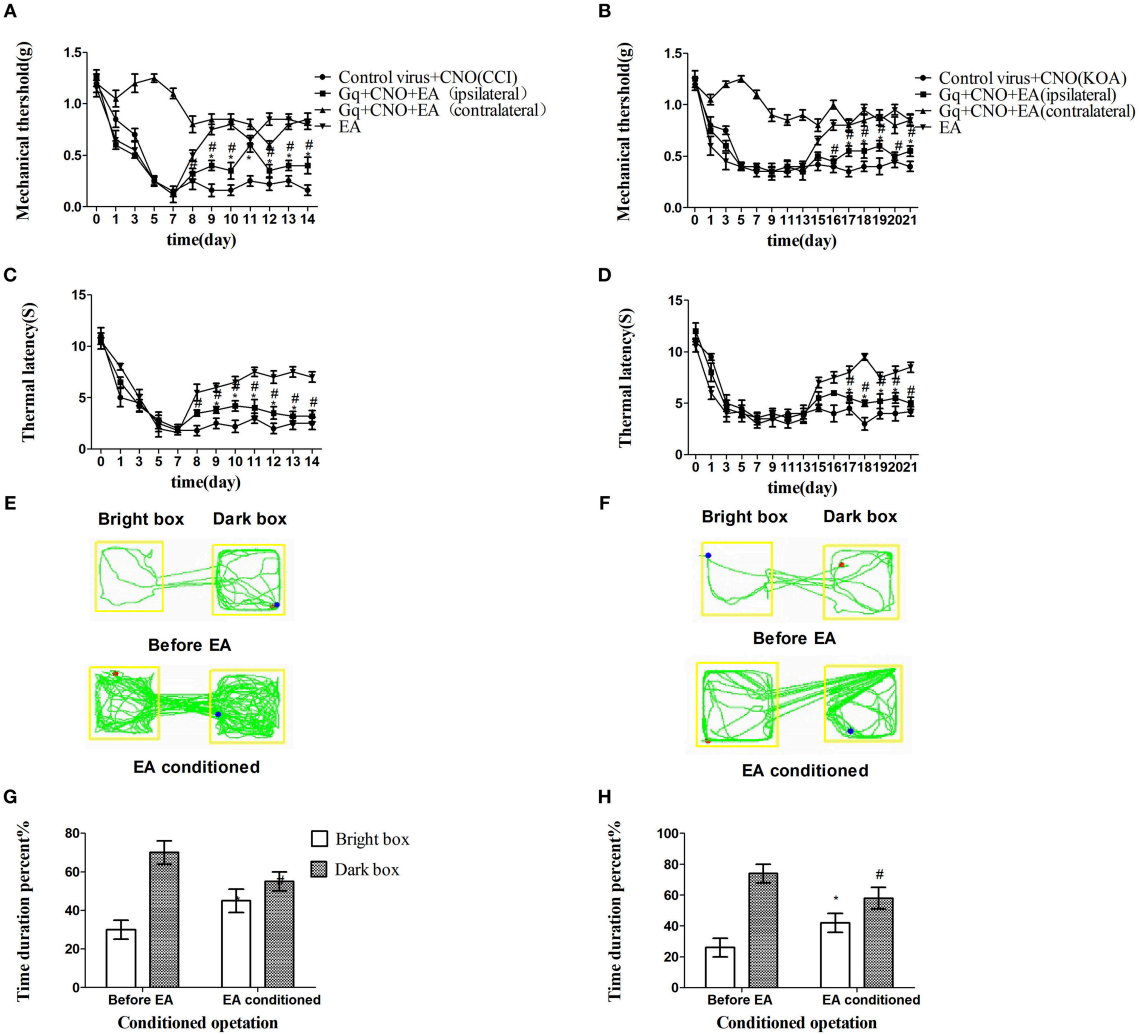
Chemogenetic activation of GABAergic neurons in the vlPAG only partly attenuated the effect of EA. **(A,B)** Time course of tactile threshold in response to von Frey filaments. **(C,D)** The noxious heat stimulus (53°C) caused a change in the thermal thresholds. Tactile **(A)** and thermal **(C)** withdrawal thresholds changes in CCI mice after chemogenetic activation. Changes in tactile **(B)** and thermal **(D)** withdrawal thresholds of KOA mice caused by chemogenetic activation. Motion map of chemogenetic activation of conditioning in the CPP test with CCI **(E)** and KOA mice **(F)**. **G,H** are summary values of motion trajectories. Virus was injected 21 days before the behavior test. CNO (1 mg/kg) was administered once a day starting from the 8th to the 14th day in the CCI model and starting from the 15th to the 21st day in the KOA model. EA (1 mA and 0.1 ms) at 2 Hz was administered for 30 min. Once a day starting from the 8th to the 14th day in the CCI model and starting from the 15th to the 21st day in the KOA model, all behavior tests were completed 2 h after CNO administration. Control groups consisted of CCI or KOA mice injected with control virus and CNO but without EA treatment. Data are expressed as means ± SEM (*n* = 12 in each group). In **A–D**, ^*^p < 0.05, compared with control group; ^#^*p* < 0.05, compared with the model group; In **G,H**,^*^p < 0.05, compared with the bright box time duration percent before chemogenetic activation; ^#^*p* < 0.05, compared with the dark box time duration percent before chemogenetic activation.

We unexpectedly found that chemogenetic activation of GABAergic neurons in the vlPAG only partly decreased the mechanical withdrawal threshold and thermal withdrawal latency in EA-treated mice (Figures 4A–D) while partly decreasing the bright box time in the CPP test (Figures 4E–H).

### The Combination of Chemogenetic Activation of GABAergic Neurons and Chemogenetic Inhibition of Glutamatergic Neurons in VlPAG Effectively Attenuated the Effect of EA

A large number of studies have shown that GABAergic neurons and glutamatergic neurons in the vlPAG play an important and complex role in nociceptive processes. Microinjection of glutamate receptor agonists or GABA antagonists into the vlPAG has a significant antinociceptive effect against noxious stimuli (Budai et al., 1998; Morgan et al., 2003). Since activation of GABAergic neurons alone in the vlPAG only partly attenuated the effect of EA, we speculated that glutamatergic and GABAergic neurons were both involved in these effects.

The rAAV-CaMKIIa-HA-KORD-IRES-mCitrine-WPRE-pA virus was applied to selectively inhibit the glutamatergic neurons of the vlPAG (Figure S4), on the basis that GABAergic neurons were excited by hM3Dq and CNO. These two systems were designed to work separately without interactions.

**Figure 5:**
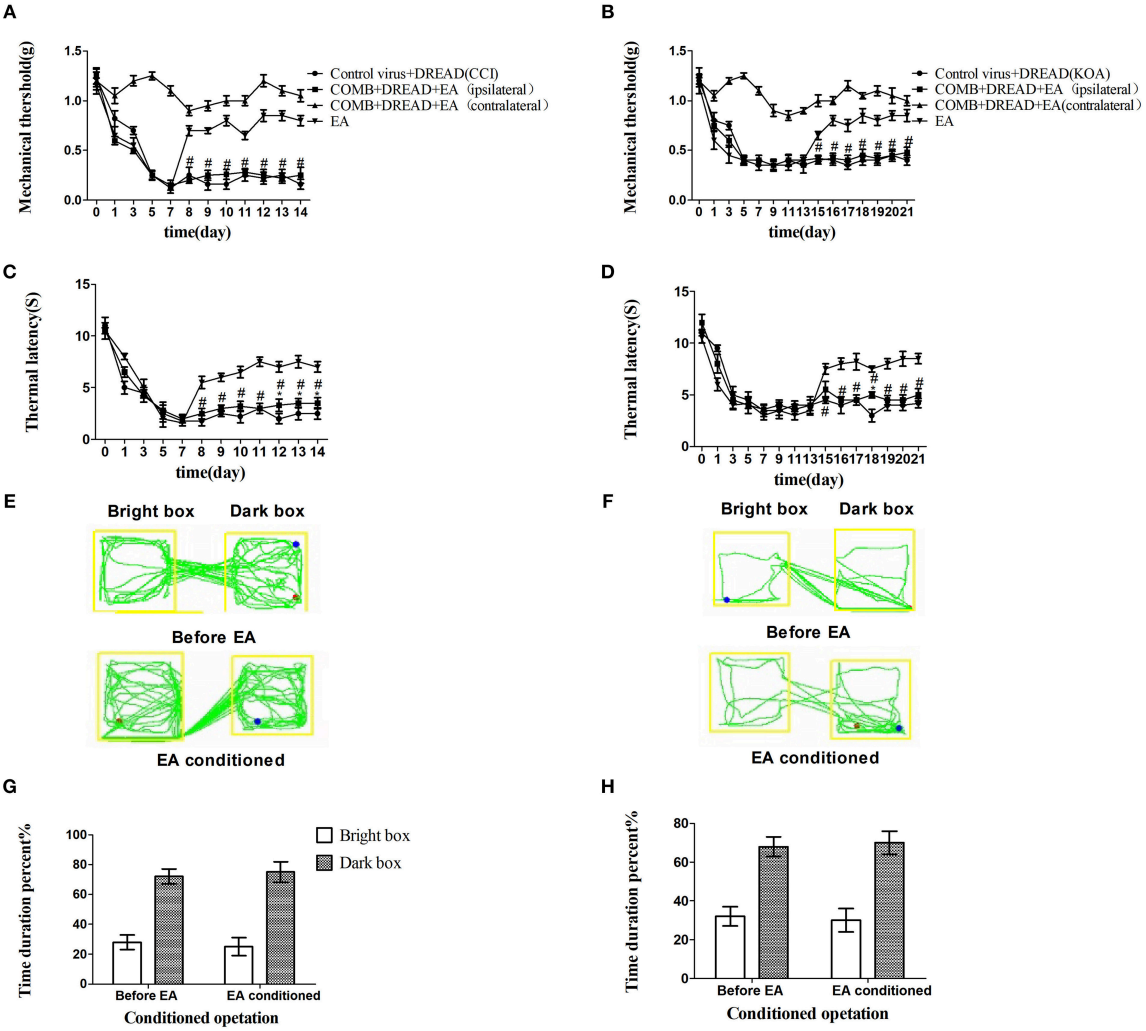
The combination of chemogenetic activation of GABAergic neurons and chemogenetic inhibition of glutamatergic neurons in the vlPAG effectively attenuate the effect of EA. COMB is abstract of combination operation. **(A,B)** Time course of tactile threshold in response to von Frey filaments. **(C,D)** The noxious heat stimulus (53°C) caused a change in the thermal thresholds. Tactile **(A)** and thermal **(C)** withdrawal thresholds changes in CCI mice after chemogenetic activation. Changes in tactile **(B)** and thermal **(D)** withdrawal thresholds of KOA mice caused by combination operation. Motion map of combination operation of conditioning in the CPP test with CCI **(E)** and KOA mice **(F)**. **G,H** are summary values of motion trajectories. Virus was injected 21 days before the behavior test. CNO (1 mg/kg) and salvinorin B (5 mg/kg) were administered once a day starting from the 8th to the 14th day in the CCI model and starting from the 15th to the 21st day in the KOA model. EA (1 mA and 0.1 ms) at 2 Hz was administered for 30 min. Once a day starting from the 8th to the 14th day in the CCI model and starting from the 15th to the 21st day in the KOA model, all behavior tests were completed 2 h after CNO administration. Control groups consisted of CCI or KOA mice injected with control virus and DREAD but without EA treatment. Data are expressed as means ± SEM (*n* = 12 in each group). In **A–D**, ^*^p < 0.05, compared with control group; ^#^*p* < 0.05, compared with the model group; In **G,H**,^*^p < 0.05, compared with the bright box time duration percent before combination operation; ^#^*p* < 0.05, compared with the dark box time duration percent before combination operation.

The combination of chemogenetic activation of GABAergic neurons and chemogenetic inhibition of glutamatergic neurons in the vlPAG powerfully decreased the mechanical withdrawal threshold and thermal withdrawal latency (Figures 5A–D) and the bright box time in the CPP test (Figures 5E–H).

### CB1 Receptors on GABAergic Neurons Is Involved in the EA Effect on Pain Hypersensitivity

Studies have found that CB1 receptors are distributed on the axon terminals of GABAergic and glutamatergic PAG neurons (Tsou et al., 1998; Vaughan et al., 2000). On this basis, it was important to explore whether the CB1 receptors on GABAergic neurons or the CB1 receptors on glutamatergic neurons participate in the analgesic action of EA.

**Figure 6:**
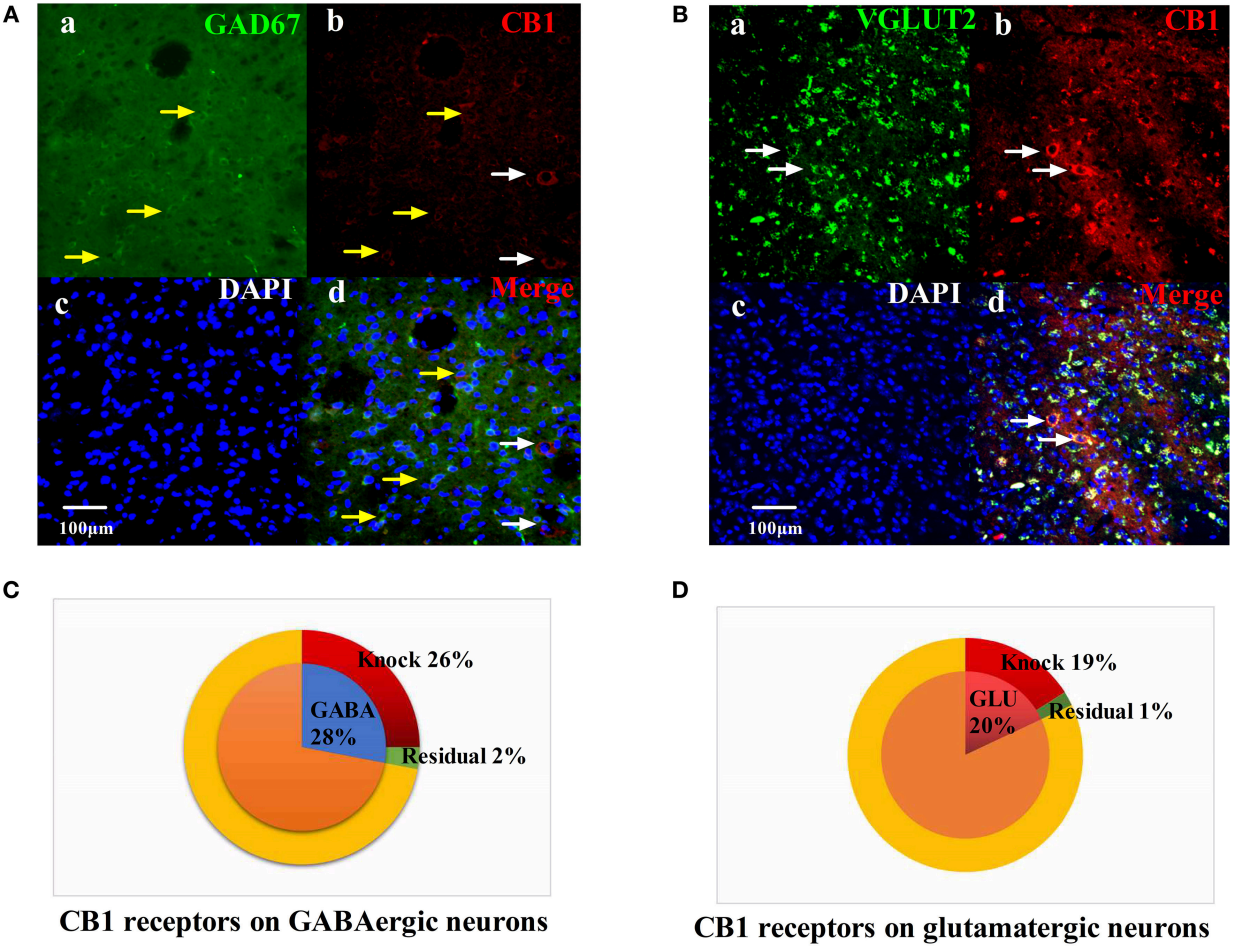
Specific knockout of the CB1 receptor on GABAergic neurons localized in the vlPAG. **(A)** rAAV-mDlx-CRE-WPRE-pA was injected into the right side of the vlPAG of mCnr1flox/flox mice. (a) GABA-immunoreactive cellsin the vlPAG (green). (b) CB1-immunoreactive cells (red). (c) DAPI nuclear staining (blue). (d) GABA-immunoreactive and DAPI nuclear staining colocalization fluorescence (purple); Yellow arrows show CB1 receptors not knocked out on GABAergic neurons. White arrows show CB1 receptors not knocked out on other large diameter neurons. Scale bar, 100 μm. **(B)** rAAV-CaMKII-CRE-WPRE-pA was injected into the right side of the vlPAG of mCnr1flox/flox mice. (a) GLU-immunoreactive cells (green). (b) CB1-immunoreactive cells in the vlPAG (red). (c) DAPI nuclear staining (blue). (d) GLU-immunoreactive and DAPI nuclear staining colocalization fluorescence (purple); White arrows show CB1 receptors not knocked out on glutamatergic neurons. Scale bar, 100 μm. **(C)** Summary data show that the percentage of CB1-immunoreactive cells was knocked out in the area of GABA-immunoreactive cells. Blue sector, GABA-immunoreactive cells; red sector, CB1-immunoreactive cells were knocked out; green sector, residual cells. **(D)** Summary data shows the percentage of CB1-immunoreactive cells knocked out in the area of glutamatergic-immunoreactive cells. Pink sector, glutamatergic-immunoreactive cells; red sector, CB1-immunoreactive cells been knocked out; green sector, residual cells. Data are expressed as the means ± SEM (*n* = 3 mice in each group).

**Figure 7:**
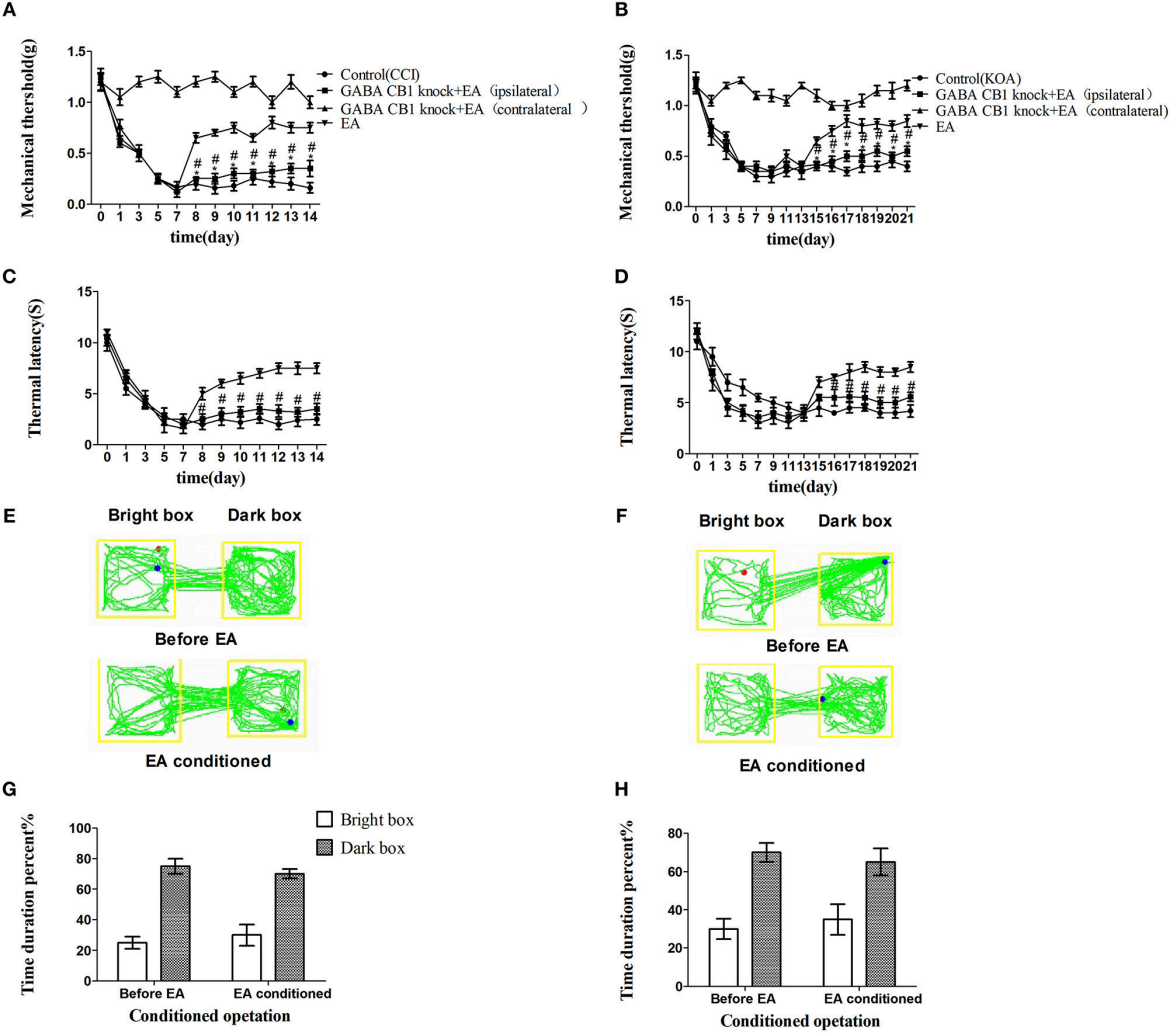
Specifically knocking out CB1 receptor of GABAergic neurons abolished the EA effect on pain hypersensitivity. **(A,B)** Time course of tactile threshold in response to von Frey filaments. **(C,D)** The noxious heat stimulus (53°C) caused a change in the thermal thresholds. Tactile **(A)** and thermal **(C)** withdrawal thresholds changes in CCI mice. Changes in tactile **(B)** and thermal **(D)** withdrawal thresholds of KOA mice. Motion map of EA conditioned CPP test of CCI **(E)** and KOA mice **(F)** on the basis of specifically knocking out CB1 receptors of GABAergic neurons. **G,H** are statistical charts of motion trajectories. The rAAV-mDlx-CRE-WPRE-pA was injected into the right side of vlPAG of mCnr1^flox/flox^ 21 days before behavior test. Same volume of saline was injected into the right side of vlPAG of mCnr1^flox/flox^ as control group. EA (1mA and 0.1ms) at 2 Hz was administered for 30 min, once a day starting from 8th day to 14th in CCI model and starting from 15th day to 21th in KOA model. Control groups are CCI or KOA mice without EA treatment. Data are expressed as means ± SEM (*n* = 5 in each group). In **A–D**, ^*^p < 0.05, compared with the control group; ^#^*p* < 0.05, compared with the model group; In **G,H**,^*^p < 0.05, compared with the bright box time duration percent before EA; ^#^*p* < 0.05, compared with the dark box time duration percent before EA.

We injected rAAV-mDlx-CRE-WPRE-pA into the right side of the vlPAG of mCnr1^flox/flox^ mice to specifically knock out the CB1 receptor on GABAergic neurons localized in the vlPAG (Figures 6A,C). Specifically knocking out the CB1 receptor on GABAergic neurons abolished the EA effect on pain hypersensitivity, as it decreased the mechanical withdrawal threshold, thermal withdrawal latency (Figures 7A–D), and the bright box time in the CPP test (Figures 7E–H).

On the other hand, rAAV-CaMKII-CRE-WPRE-pA was injected into the right side of the vlPAG of mCnr1^flox/flox^ mice for the purpose of specifically knocking out the CB1 receptor on glutamatergic neurons localized in the vlPAG (Figures 6B,D) and only slightly decreased the EA effect on pain hypersensitivity (Figures S3A–H). It seems that the CB1 receptor on GABAergic neurons localized in the vlPAG was the basis of the EA effect on pain hypersensitivity.

## Discussion

The vlPAG, as an essential part of the neural pathway that mediates pain sensation, has been extensively studied (Vaughan et al., 1997; Ho et al., 2013; Tovote et al., 2016). Consistent with these studies, we found that inhibition of vlPAG neurons by chemogenetics can produce significant antinociceptive effects. We also found that chemogenetic activation of vlPAG neurons resulted in noxious hypersensitivity, and the results of this two-way manipulation were consistent with the bidirectional regulation of the vlPAG in nociceptive regulation (Koutsikou et al., 2015; Hernandez-Leon et al., 2016; Samineni et al., 2017). The vlPAG is critical for the mechanisms of EA-induced analgesia, and we have demonstrated in previous work that during KOA chronic pain, EA exerted an analgesic effect by increasing the levels of CB1 receptors and 2-AG in the vlPAG that had been significantly reduced (Yuan et al., 2018). In this study, we provided new experimental evidence that chemogenetic inhibition of GABAergic neurons in the vlPAG was able to replicate the antinociceptive effect of EA and accordingly further verify that the vlPAG is essential for EA analgesia.

**Figure 8:**
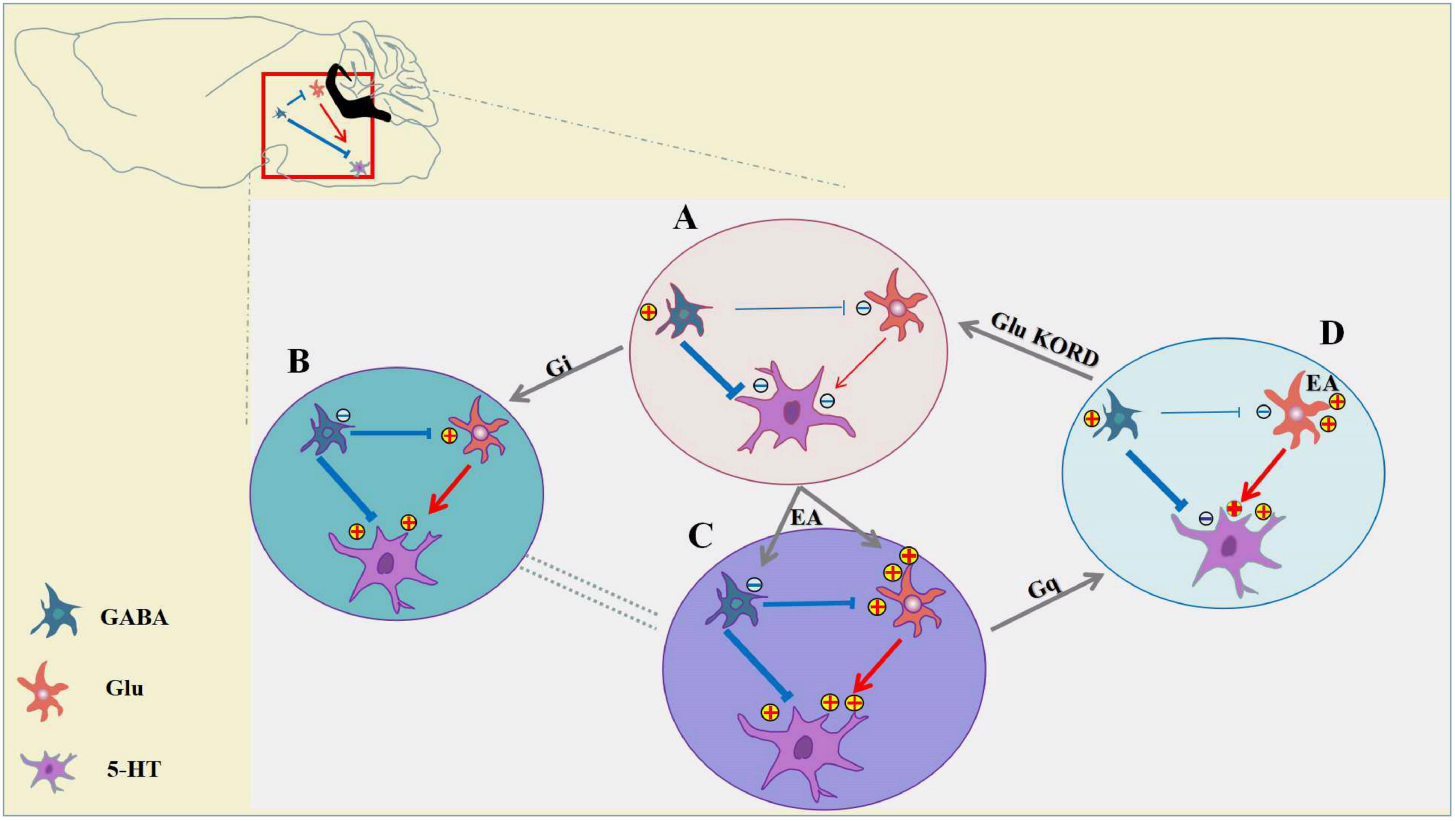
Inhibition of GABAergic neurons and excitation of glutamatergic neurons in the vlPAG participate in EA analgesia. **(A)** Under pain conditions, GABAergic neurons are excited, resulting in glutamatergic, and serotoninergic neuron inhibition. **(B)** Chemogenetic inhibition of GABAergic neurons results in activation of glutamatergic neurons and serotoninergic neurons, and the descending inhibitory pain pathway would be activated to relieve the pain. **(C)** EA synchronously inhibits GABAergic neurons and activates glutamatergic neurons, thereby allowing serotoninergic neurons to be excited sufficiently. **(D)** With chemogenetic inhibition of GABAergic neurons, glutamatergic neurons are still activated by EA, and serotoninergic neurons in the descending inhibitory pain pathway remained partially activated. Chemogenetic activation of GABAergic neurons and inhibition of glutamatergic neurons is sufficient to attenuate the effects of EA.

The cellular mechanisms of analgesia and hyperalgesia, which involve in inhibitory and excitatory neurotransmission in the vlPAG have not been directly evaluated. It is not known how distinct neuron subpopulations in the vlPAG are engaged in the descending pain modulation pathway. For the first time, we showed that chemogenetic inhibition of GABAergic vlPAG neurons produced antinociceptive effects, while the combination of chemogenetic activation of GABAergic neurons and chemogenetic inhibition of glutamatergic neurons in vlPAG effectively attenuated the effect of EA. It is hypothesized that GABAergic neurons have a tonic inhibitory effect on vlPAG glutamatergic neurons, while glutamate neurons are output neurons that project to the rostral ventromedial medulla (RVM) (Vaughan et al., 1997; Morgan et al., 2008; Ho et al., 2013). As we can see from Figure 8, chemogenetic inhibition of GABAergic neurons in the vlPAG also produces activation of glutamatergic neurons according to the GABA disinhibition hypothesis, whereas selective activation of GABAergic neurons is not sufficient to attenuate the effect of EA on the condition that some glutamatergic neurons are still directly activated by EA (Figure 8C). This is the potential reason why specific chemogenetic inhibition of GABAergic neurons is sufficient to simulate the EA effect, while the reverse of the EA effect requires the combination of chemogenetic activation of GABAergic neurons and chemogenetic inhibition of glutamatergic neurons in the vlPAG. More studies are necessary to determine the neural circuitry of the vlPAG and connectivity with other brain regions. It is also possible that that GABAergic and glutamatergic neurons in the vlPAG have local circuits, such as synaptic connections between GABAergic neurons and glutamatergic neurons in the vlPAG. Future studies should examine the exact physiological function of GABAergic and glutamatergic neurons in the vlPAG local circuits and through synaptic connections between brain regions.

Endogenous cannabinoid ligands and CB1 receptors are widely present in the nociceptive and descending inhibitory pathways (Mitrirattanakul et al., 2006). CB1 receptors are expressed in both nerve endings of GABAergic neurons and glutamatergic neurons in the PAG, which provides histological evidence that the activation of the CB1 receptor may regulate GABAergic and glutamatergic neurotransmission (Drew et al., 2009; Hu et al., 2014). WIN55212-2 (WIN), a CB1R agonist, modulates various K^+^ and Ca^2+^currents that could activate GABAergic and glutamatergic neurons (Straiker et al., 1999; Yazulla et al., 2000; Fan and Yazulla, 2003; Straiker and Sullivan, 2003; Yazulla, 2008; Schwitzer et al., 2016).

Consistent with previous electrophysiology results, we experimentally confirmed that the CB1 receptors on GABAergic and glutamatergic neurons are involved in the analgesia of EA. Using the Cre/loxP system, we are able to specifically knock out the CB1 receptor on GABAergic neurons or glutamatergic neurons localized in the vlPAG. Knocking out the CB1 receptor on GABAergic neurons abolished most of the EA effect on pain hypersensitivity, while knocking out the CB1 receptor on glutamatergic neurons localized in the vlPAG, only lessened some of the EA effect on pain hypersensitivity. According to our results, EA can simultaneously inhibit GABAergic neurons and excite glutamatergic neurons in the vlPAG via CB1 receptors. Chemogenic CB1 knock-out on GABAergic neurons abolished the effect of EA while chemogenic activation of GABAergic neurons only partly attenuated the effect of EA. It suggested that the CB1 receptors on GABAergic neurons may also be required for activation of the CB1 receptor on glutamatergic neurons localized in the vlPAG, which conforms to the theory of ON- and OFF-cells in the vlPAG (Heinricher et al., 1987). According to Wang's research (Wang et al., 2016), a CB1R agonist reduced the mIPSC frequency regardless of whether AMPA receptors were blocked, while it affected the mEPSC frequency when inhibitory inputs were present. It indicated that the activation of GABAergic neurons may be necessary for glutamatergic neuron excitation via CB1 receptors.

Through chemical genetic manipulations, we have observed for the first time that EA can exert an analgesic effect by simultaneously inhibiting GABAergic neurons and stimulating glutamatergic neurons. We know that when pain occurs, it is accompanied by a functional imbalance between GABAergic neurons and glutamatergic neurons. The relative balance of GABAergic neurons and glutamatergic neurons is critical for the maintenance of homeostasis (Siegfried and de Souza, 1989; Schmidtko et al., 2008). The microinjection of GABA_A_ receptor antagonists or glutamate agonists into the vlPAG produces antinociceptive effects (Bobeck et al., 2009; Liao et al., 2011). According to recent research by Vijay K, activation of glutamatergic neurons or inhibition of GABAergic neurons by chemical genetics achieves an effective inhibition of nociceptive sensation, in spite of that experiment being performed in normal mice rather than in a mouse pain model, as in the present experiments (Samineni et al., 2017). Despite the support of those experiments, the effect of clinical application of GABA_A_ receptor antagonists or glutamate agonists is not optimistic (de Meij et al., 2014; Bruhn et al., 2017). In clinical treatment, it is indeed difficult to find a drug that can simultaneously act on both GABAergic and glutamatergic neurons, as they belong to different neuroreceptor systems with two distinct functions. According to our results, EA may simultaneously inhibit GABAergic neurons and activate glutamatergic neurons in the vlPAG via CB1 receptors to exert antinociceptive effects. The results of this study provide experimental evidence contributing to our understanding of the dual target adjustment mechanism of EA analgesia and homeostasis recovery under pain conditions.

In conclusion, we have demonstrated a novel role of GABAergic neurons and glutamatergic neurons in the vlPAG in EA antinociceptive effects. EA can simultaneously inhibit GABAergic neurons and excite glutamatergic neurons in the vlPAG via CB1 receptors, thereby exerting antinociceptive effects. Knocking out the CB1 receptor on GABAergic neurons is sufficient to abolish the EA effect on pain hypersensitivity, while the combination of activation of GABAergic neurons and inhibition of glutamatergic neurons in vlPAG is sufficient to attenuate the effect of EA. EA synchronously regulates the GABAergic system and the glutamatergic system with distinct functions through the CB1 receptor. This finding may provide new ideas for the clinical treatment of pain and the development of analgesic drugs.

## Author Contributions

J-QS and ML designed this experiment. HeZ mainly completed this experiment. H-CX, H-PL, L-XL, and X-FH provided help in brain stereotactic injection work. HoZ, W-YM, LL, CC, YS, and R-YZ assisted in the completion of animal behavior experiments. PZ, J-QS, and ML provided help in writing and modifying this paper.

### Conflict of Interest Statement

The authors declare that the research was conducted in the absence of any commercial or financial relationships that could be construed as a potential conflict of interest.
