# Supplementary material for: Inhibition of GABAergic Neurons and Excitation of Glutamatergic Neurons in the Ventrolateral Periaqueductal Gray Participate in Electroacupuncture Analgesia Mediated by Cannabinoid Receptor
Source: Front Neurosci. 2019 May 17;13:484. doi: 10.3389/fnins.2019.00484 (PMC6533898; doi:10.3389/fnins.2019.00484)
Supplement: Supplementary file 1 [file Data_Sheet_1.PDF]

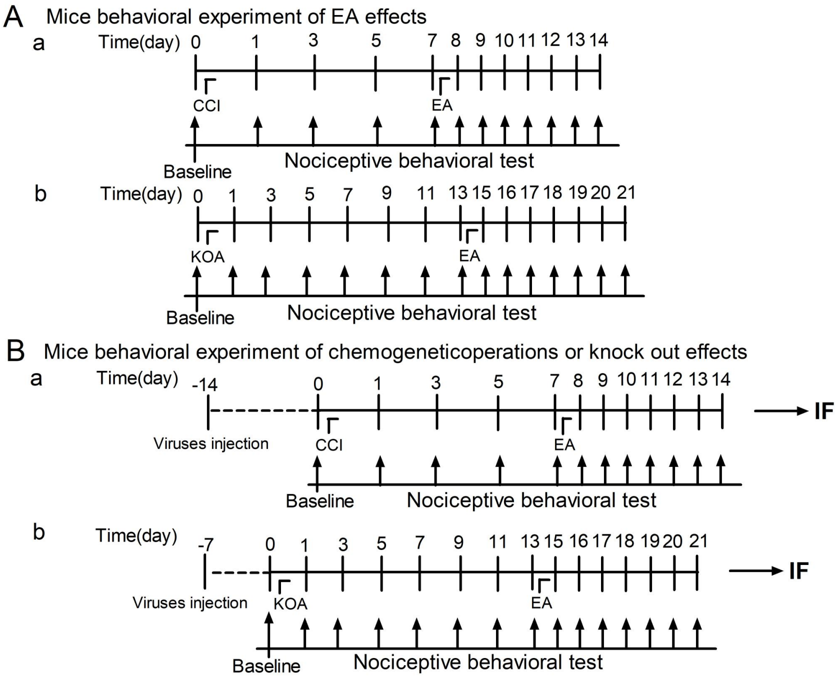

Fig. S1 Experimental design timeline. Abbreviations: CCI, Chronic constriction injury model; KOA, Knee osteoarthritis model; EA, Electroacupuncture; IF, Immunofluorescence labeling. Nociceptive behavioral tests of EA effect in CCI and KOA mice(A). Nociceptive behavioral tests were performed before (baseline) model induction. In the CCI group(a), Nociceptive behavioral tests were performed at 1, 3, 5, 7 d after the model induction; Nociceptive behavioral tests were performed from 8 -10 d after EA treatment. In the KOA group(b), Nociceptive behavioral tests were performed at 1, 3, 5, 7, 9, 11, 13 d after the model induction; Nociceptive behavioral tests were performed from 15 -21 d after EA treatment. B. In CCI group(a), viruses were injected into vlPAG of mice 14 days before model induction. In KOA group(b), viruses were injected into vlPAG of mice 7 days before model induction. The nociceptive behavioral tests were similar to (A)

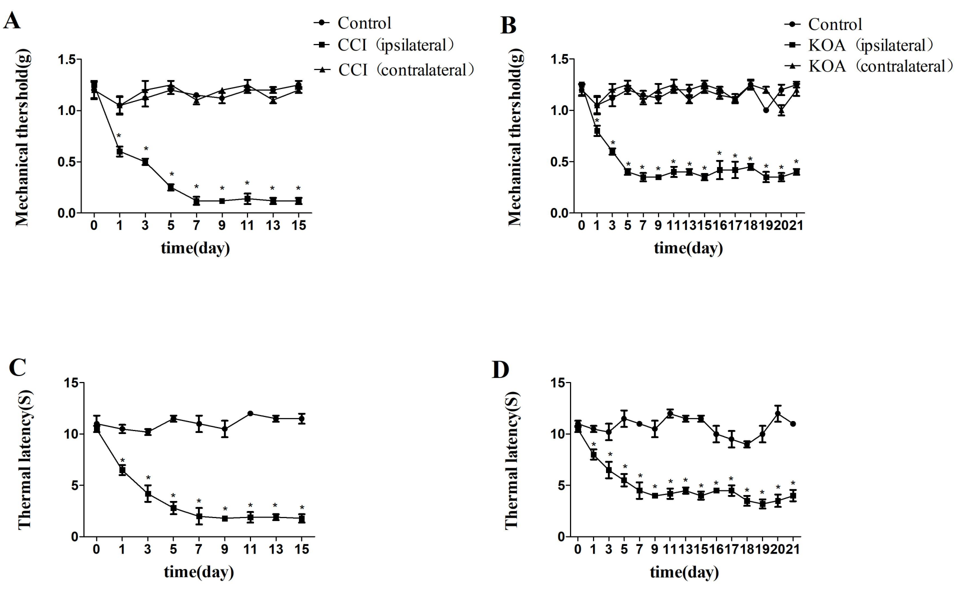

Fig. S2 The change of tactile threshold and thermal withdrawal latency in CCI and KOA mice. (A, B) Time course of tactile threshold in response to von Frey filaments. (C, D) The noxious heat stimulus (53°C) caused change of thermal thresholds . The change of tactile (A) and thermal (C) withdrawal thresholds of CCI mice. The change of tactile (B) and thermal (D) withdrawal thresholds of KOA mice. Control groups are sham operation mice. Data are expressed as means ± SEM (n = 12 mice in each group). $^{*}p < 0.05$, compared with control group.

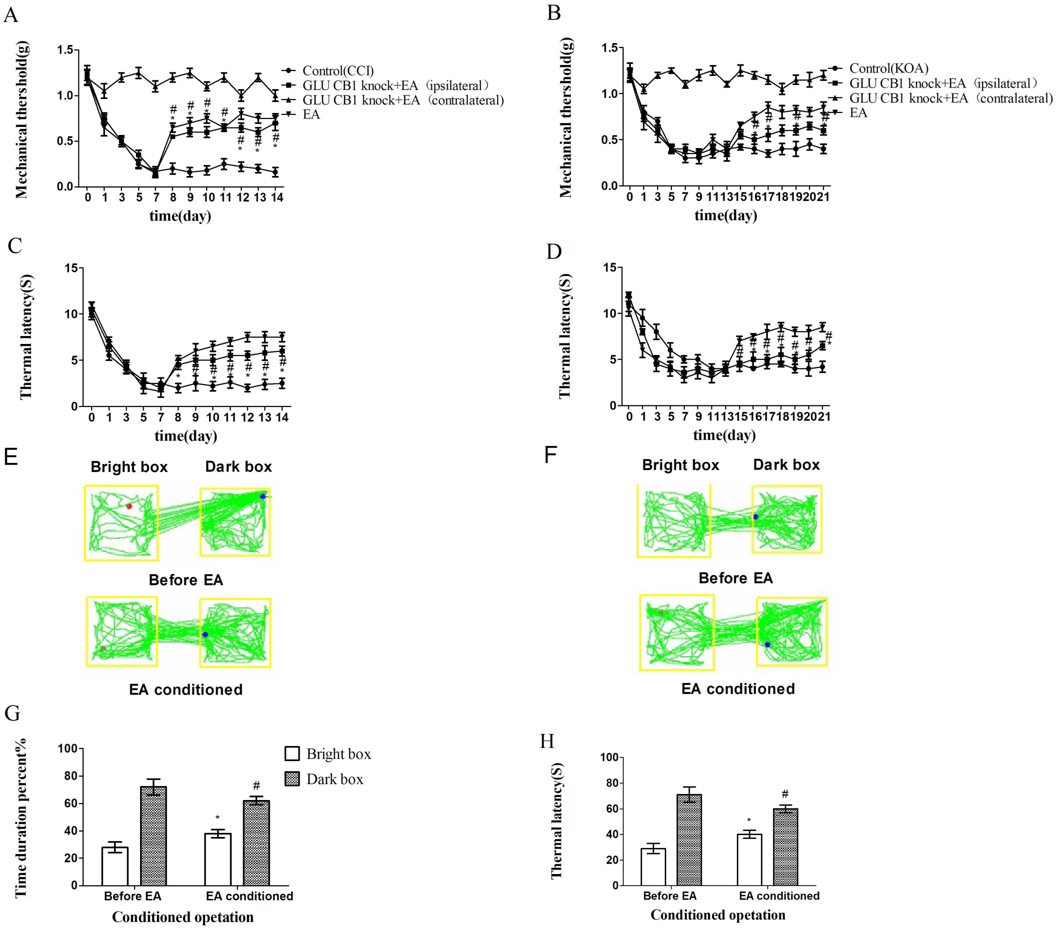

Fig. S3 Specifically knocking out CB1 receptor of glutamatergic neurons locolized in the vlPAG only decrease little part of EA effect on pain hypersensitivity. (A, B) Time course of tactile threshold in response to von Frey filaments. (C, D) The noxious heat stimulus (53°C) caused a change in the thermal thresholds. Tactile (A) and thermal (C) withdrawal thresholds changes in CCI mice. Changes in tactile (B) and thermal (D) withdrawal thresholds of KOA mice. Motion map of EA conditioned CPP test of CCI (E) and KOA mice (F) on the basis of specifically knocking out CB1 receptor of glutamatergic neurons. (G) and (H) are statistical charts of motion trajectories.The rAAV-CaMKII-CRE-WPRE-pA was injected into the right side of vlPAG of mCnr1$^{flox/flox}$ 21 days before behavior test. Same volume of saline was injected into the right side of vlPAG of mCnr1$^{flox/flox}$ as control group. EA(1mA and 0.1ms) at 2 Hz was administered for 30 min, once a day starting from 8th day to 14th in CCI model and starting from 15th day to 21th in KOA model. Control groups are CCI or KOA mice without EA treatment. Data are expressed as means ± SEM (n =5 in each group). In figure (A-D), $^{*}p< 0.05$, compared with the control group; $^{\#}p< 0.05$, compared with the model group; In figure (G) and (H),$^{*}p< 0.05$, compared with the bright box time duration percent before EA; $^{\#}p< 0.05$, compared with the dark box time duration percent before EA .

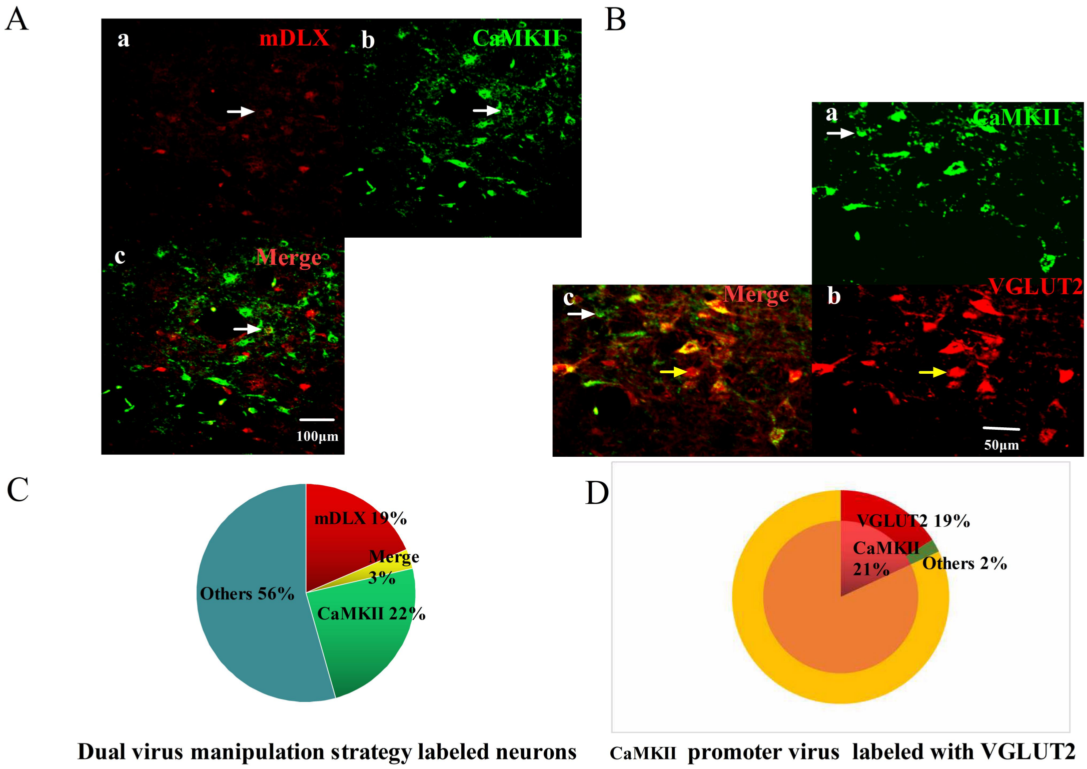

Fig S4 Dual virus manipulation strategy labeled neurons. (A) Dual virus （mDLX and CaMKII）manipulation strategy labeled neurons. (a) mDLX promoter virus labeled cells in the vlPAG (red). (b)CaMKII labeled neurons in the vlPAG(green). (c) Merge of dual virus manipulation strategy labeled neurons(yellow); White arrows show the merge neurons.Scale bar, 100 μm. (B) CaMKII promoter virus labeled with VGLUT2. (a) CaMKII promoter virus labeled cells in the vlPAG (green). (b)VGLUT2 labeled neurons in the vlPAG(red). (c) Merge of co-labeled neurons(yellow); White arrows show the CaMKII promoter virus labeled neurons not merge with VGLUT2 labeled neurons; Yellow arrows show the VGLUT2 labeled neurons not merge with CaMKII promoter virus labeled neurons.Scale bar, 50 μm. (C) Summary data show that the percentage of merge of dual virus manipulation strategy labeled neurons. Red sector, mDLX promoter virus labeled cells ; green sector, CaMKII promoter virus labeled neurons;Yellow sector, co-labeled neurons. (D) Summary data show that the

percentage of CaMKII promoter virus labeled with VGLUT2. Pink sector, CaMKII promoter virus labeled cells ;Red sector, VGLUT2 labeled neurons; Green sector, co-labeled neurons. Data are expressed as the means±SEM (n =3 mice in each group).

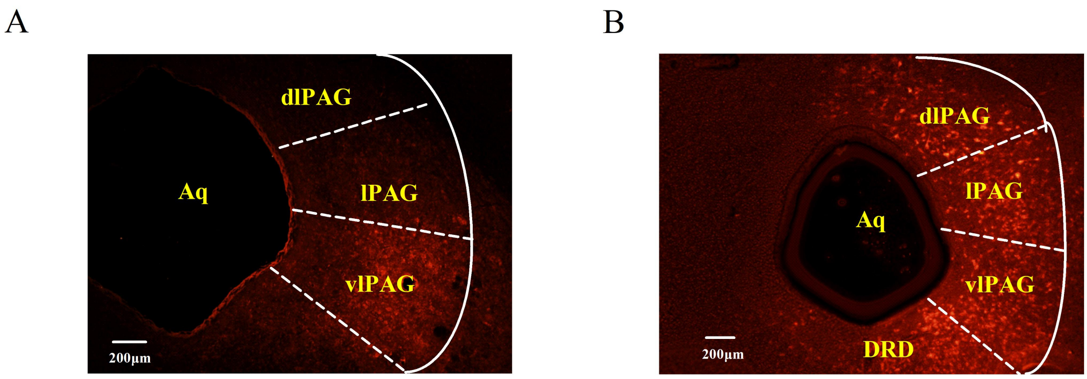

Fig S5 Inclusion and exclusion of viral infection site and infected area. (A)Viral injection site and infected area in vlPAG, included in statistics ;(B) Viral injection site and infected area not in vlPAG, exclusion of statistics analysis ;
